# Can acid produced from probiotic bacteria alter the surface roughness, microhardness, and elemental composition of enamel? An in vitro study

**DOI:** 10.1007/s10266-023-00804-1

**Published:** 2023-03-30

**Authors:** Swagata Saha, Aditi Chopra, Shobha Ullas Kamath, Namita N. Kashyap

**Affiliations:** 1 Department of Periodontology, Manipal College of Dental Sciences, Manipal, Manipal Academy of Higher Education, Manipal, 576104 India; 2 Department of Biochemistry, Kasturba Medical College, Manipal, Manipal Academy of Higher Education, Manipal, 576104 India

**Keywords:** Probiotics, Teeth, Enamel, Caries, Lactobacilli, Demineralization, Oral health, Erosion, Critical pH, Lactic Acid, Bifidobacterium

## Abstract

Probiotics are live microorganisms that upon administration in adequate amounts provide various health benefits to the host. Probiotics are “lactic acid-producing bacteria” as they release large amounts of organic acids, particularly lactic acids, in their surrounding environment. Although the acids produced by probiotics are beneficial for gastrointestinal and vaginal health, the acidogenic nature of probiotics has raised concerns among dental professionals, especially concerning their effect on the enamel and dentin. Previous studies have found that probiotics can lower the pH of the saliva and cause essential elements like Calcium and Phosphorus to leach from the enamel. This can alter the surface topography of enamel and increase the risk of enamel defects. Studies have also noted that probiotic bacteria can replace cariogenic bacteria and lower the risk of tooth decay. However, the effect of acid produced by probiotics on the enamel surface remains unclear. Hence, the present study aims to evaluate the effect of probiotics on the surface roughness, microhardness, and elemental composition of enamel compared to 0.1 M Lactic acid (demineralizing agent). Twenty enamel sections were randomly divided into groups and subjected to a pH cycling model using a probiotic suspension and 0.1 M lactic acid. The changes in the surface roughness, microhardness, surface morphology, and elemental composition of the enamel with regard to Carbon, Oxygen, Sodium, Hydrogen, Magnesium, Phosphorus, Fluoride, Chlorine, and Calcium of the enamel were evaluated before and after the emersion in both the groups. The results showed a significant increase in the mean surface roughness in the probiotic group before and after the exposure. The microhardness of the enamel decreased along with altered arrangement of the enamel prisms, increased striations, scratch marks, and pitting after exposure to the probiotic group. A decrease in the atomic/weight% for Calcium, Phosphorous, Fluoride, Aluminium, and Oxygen and an increase in the weight/atomic% for Carbon, Nitrogen, and Sodium were noted compared to the baseline in the probiotic solution. The results in the probiotic group were comparable to the 0.1 M lactic acids. The pH changed from 5.78 to 3.06 at the end of 24 h in the probiotic group. Based on these findings, we conclude that exposure to probiotics can affect microhardness and surface roughness and cause leaching of essential elements like Calcium and Phosphorous from the enamel.

**Supplementary Information:**

The online version contains supplementary material available at 10.1007/s10266-023-00804-1.

## Introduction

Probiotics are live microorganisms that upon administration in adequate amounts provide various health benefits to the host [1]. Currently, probiotics are the third most commonly consumed dietary supplement and the most popular superfood after vitamins and minerals. According to 2012 National Health Interview Survey [NHIS], around four million US adults and 300,000 children consumed probiotics in one month [2, 3]. Probiotic formulations are available in various forms like lozenges, tablets, powdered sachets, yoghurt, chewing gums, milk, liquid syrups, and dietary supplements [3].

Probiotics formulations have a high number of live bacteria such as *Lactobacillus, Bifidobacterium, Saccharomyces, Enterococcus, Bifidobacterium Streptococcus, Pediococcus, Leuconostoc, *and* Bacillus* [3–5]. These microorganisms are classified as “lactic acid-producing bacteria”, as they release large amount of organic acids, particularly lactic acid, acetic acid, butyric acid, citric acid, hippuric acid, orotic acid, uric acid, pyruvic acid, and succinic acid in their environment [4–7]. This acidogenic nature of probiotic bacteria is beneficial for treating various systemic diseases, particularly disorders of the gastrointestinal, vaginal, urogenital, and oropharyngeal regions [8]. However, the increased acidic production has raised concerns among dental professionals, as many studies have questioned the effect of acid produced by probiotic bacteria on the pH of the saliva and how it can affect the ecology of the oral microbiome and demineralization of enamel surface [9–14].

The pH or acidity of the oral cavity is a key environmental factor that affects the nature, physiology, ecology, demineralizing potential, and pathogenicity of the oral microflora [15–17]. The pH regulates the growth, diversity, and survival of many oral bacterial species [20]. When the pH falls below 5.5, many acid-tolerant bacteria such as *Lactobacillus, Prevotella, Propionibacterium,* and *Streptococcus* species start proliferating compared to other members of the biofilm. This causes a loss of microbial diversity and increases the growth of cariogenic species [20–23]. In an acidic environment, where there is high lactic acid concentration, the species such as *Granulicatella adiacens, Lautropia, Neisseria, Streptococcus, and Veillonella* bacteria are found to present in high numbers [16]*.* Based on the recent integrative plaque hypothesis, changes in the acidity or alkalinity of the saliva is one of the major drivers of enamel demineralization and caries [20]. Since probiotic bacteria are acidogenic in nature produce, few studies have linked them to the development of “carious and erosive lesions” on the tooth [9–22]. Probiotic bacteria such as “*Lactobacilli* and *Bifidobacterium”* can survive in acidic environment such as from initial soft or deep carious lesions and infected dentin [21–24]. This is attributed to the ability of probiotic bacteria to maintain their pH against extracellular acidification, have strong cell membrane durability, and the ability to produce intracellular alkaline to survive. Matsumoto et al., 2005, observed that *Lactobacillus salivarius,* a common probiotic bacterium, can establish itself with other members of the oral biofilm. It has the potential to induce caries even in the absence of *Streptococcus mutans* [25]. On the contrary, there are studies that popularize the beneficial effect of probiotics on the ground that it can replace cariogenic bacteria and reduce the risk of caries [22–27].

However, one should note that even if probiotic can replace cariogenic bacteria, the effect of acids produced by probiotics on the enamel surface and their role in tooth demineralization should be explored. A recent systematic review and meta-analysis by Hao et al. [2021] [9] also stated that probiotics did not cause any significant reduction in cariogenic species in saliva of individuals consuming probiotics [9]. The review also has raised concerns regarding the acidogenic nature of probiotics and the need for further studies to establish their safety. To our knowledge, no study has evaluated the change in the elemental composition, surface roughness, microtopography, and microhardness of enamel by probiotics. Thus, the present study aims to evaluate whether exposure to probiotics has any effect on the surface roughness, microhardness, and elemental composition of enamel. The main objectives of the study are to evaluate and compare the changes in the following aspects before and after exposure to probiotics compared to the standard demineralizing agent (0.1 Lactate) using the pH cycle regime:

1. Changes in the surface morphology of the enamel
2. Changes in the elemental composition of enamel, i.e. Carbon [C], Oxygen [O], Sodium [Na], Hydrogen [H], Magnesium [Mg], Phosphorus [P], Fluoride [F], Chlorine [Cl], Calcium [Ca], and Nitrogen [N]
3. Surface roughness [Ra] and microhardness of the enamel
4. Changes in the pH of the probiotic solution at 2, 4, 6, 8, 12, and 24 h.

## Materials and methods

20 orthodontically extracted premolar teeth with complete root ends were collected from subjects aged above 18 years of age. Post-extraction, the teeth were immediately washed with sodium hypochlorite solution and scaled with Gracey curettes and machine-driven ultrasonic scalers. This was done to remove all the hard and soft tissue deposits and tissue tags adhering to the tooth. The teeth were checked for caries, cracks, fracture, attrition, abrasion, erosion, and signs of hypo-calcification by examining them under Stereo Microscope [Cartoon Optimal Industries Ltd, SCW-X model] at 40X magnification. Teeth with any of the above signs were excluded. The selected teeth were then stored in saline at 4 °C, with the addition of thymol crystal dissolved in 4 mL of alcohol to prevent the growth of bacteria till in use [28, 29].

**Fig. 1:**
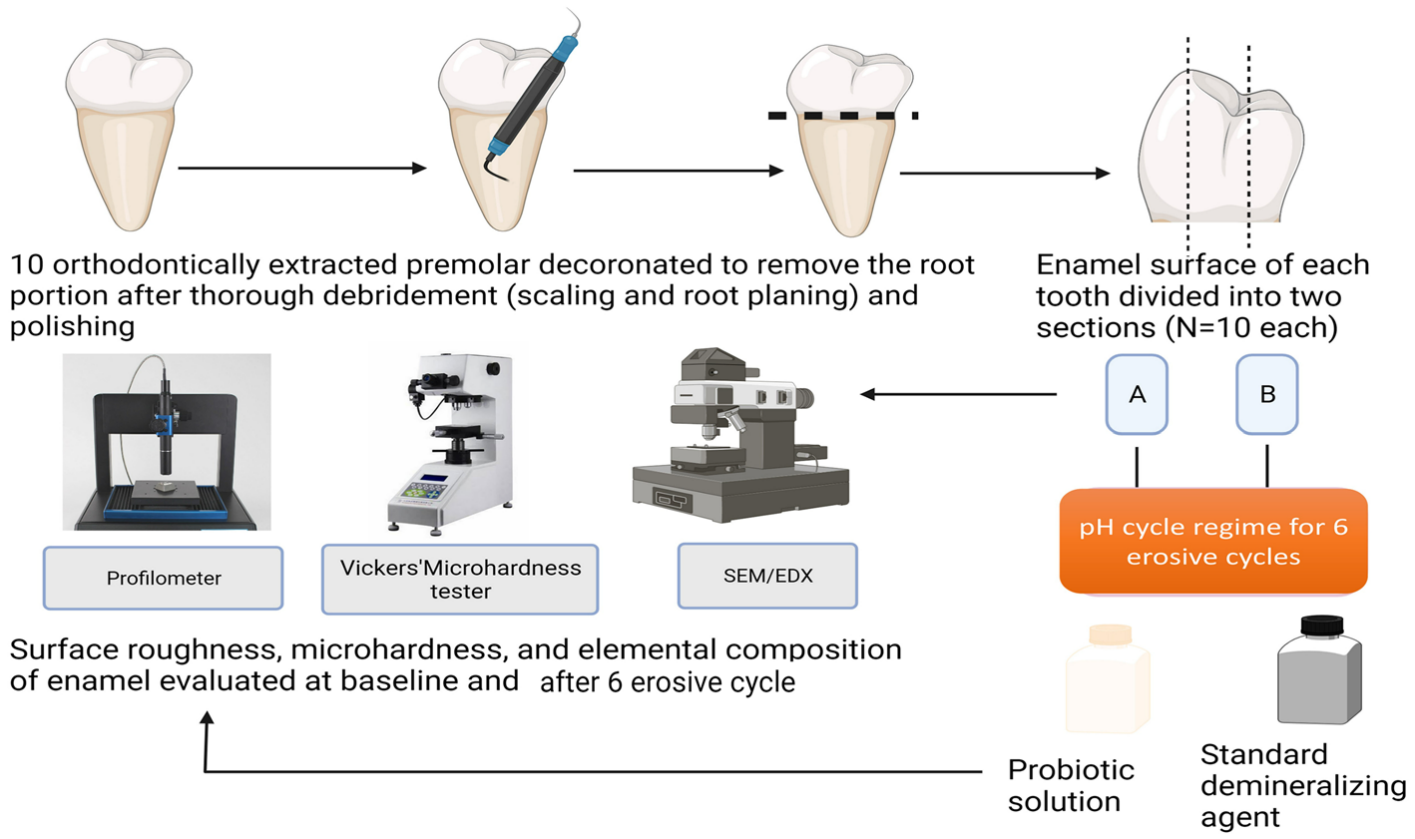
Schematic representation of the methodology (created in Biorender)

All the teeth were subsequently sectioned at the cementoenamel junction in a mesiodistal direction using a low-speed micromotor and diamond disc bur to separate the crown and root portion. The enamel was carefully separated and placed on a paper sticker. The paper sticker was placed on a mixing slab with the sticker surface facing upward, and the separated enamel was affixed to the paper. All the enamel samples were sequentially sanded with sandpaper numbers 800, 1500, and 2000. After sanding, the specimens were polished with alumina and a rubber cup with a low-speed micromotor [Fig. 1]. The buccal surface of each tooth was subdivided into 3 sections of 3 mm X 3 mm each [Fig. 1]. All the sections from tooth number 1 were labelled as 1A, 1B, and 1C. The sections of the remaining teeth were labelled similarly. The following baseline analysis was done for all the enamel sections:

1. *SEM analysis for evaluating the surface morphology and elemental analysis* for Carbon [C], Oxygen [O], Sodium [Na], Hydrogen [H], Magnesium [Mg], Phosphorus [P], Fluoride [F], Chlorine [Cl], Calcium [Ca], Aluminium [Al], Nitrogen [N], and Sulphur [S] was done for all the sections using scanning electronic microscopy [SEM] with Energy dispersive X-ray [EDX] spectroscopy without spluttering [Zeiss EVO MA 18 with Oxford EDS] at the magnification from 500X, 1000X, and 2000X before the exposure as described previous studies [30].
2. *Surface roughness [Ra] was measured by a surface profilometer* [Surtronic 3 + profilometer, Rank Taylor Hobson, Leicester, UK] [31]. Three readings from three different points from each enamel section to minimize error. The baseline tracings were carefully marked and post-experimental tracing was performed at the same location as the baseline.
3. *Microhardness was determined using Vicker’s microhardness tester* [Matsuzawa Microhardness testing machine [MMT – X7A, Japan]] under 100 g load for 5 s. The samples were rinsed in water and dabbed dry with absorbent paper before calculating the baseline values. The test specimens were stabilized on the stage of the tester. The area to indent was determined by focusing with 10 × lens. After this, the test was carried out at the site of marked indentations with a rate of 100 g load for 10 s perpendicular to the enamel surface with the diamond indenter. The indentation formed was viewed and measured on the display monitor with a10 × objective lens and an in-built computerized program. The average microhardness of the specimen was determined from three indentations to avoid any discrepancy since the enamel surface has a curvature. An average of the three readings was calculated per sample [32].

After obtaining the baseline readings, all the enamels sections were randomly divided into two groups as follows:

1. Group 1 [Enamel section exposed to a probiotic solution, *N* = 10]: The probiotic solution was prepared by dissolving the one packet of Darolac sachet [Aristo Pharmaceuticals Pvt Ltd in India] in 30 ml of boiled and cooled distilled water [as per the manufacturers instruction]. The microbial content of 1 g of probiotic was found to contain *Lactobacillus rhamnosus (360.5.10*^*6*^* CFU), Lactobacillus acidophilus (662.5.10*^*6*^* CFU), Bifidobacterium longum (80.5.10*^*6*^* CFU), *and* Saccharomyces boulardii (137.5.10*^*6*^* CFU)* as measured by the optical density calculations*.* This solution was mixed with artificial saliva in a ratio of 1:1 as described previously [33, 35]. The artificial saliva was formulated by dissolving Albumin, Potassium Phosphate, Sodium Fluoride, and Methyl Cellulose in a small amount of water. Magnesium Chloride, Dextrose, and 0.2 mg/mL Methylparaben were dissolved in warm water and cooled down before mixing the two solutions. The final composition of the solution was 0.1% Albumin, 0.75% Methyl Cellulose, 0.034% Potassium Phosphate, 0.01% Sodium Fluoride, 0.2% Methylparaben, 0.062% Magnesium Chloride, 4.69% Dextrose, and Gelatin as flavouring agent. 20 μl of this solution was put into a 10 mL volumetric flask and then diluted with water. Approximately 0.1 mM of glucose was added, to simulate the basal concentration of glucose that remains in the oral cavity at a given time (34).
2. Group 2 [Enamel section exposed to a probiotic solution, *N* = 10]: Enamel was exposed to a standard demineralizing agent mixed with artificial saliva in the ratio of 1:1. The demineralizing solution was prepared as follows: 6% wt carboxymethyl cellulose gel, 0.1 M Lactic acid, and 1 M Potassium Hydroxide [35].

## pH cycling regimen

The enamel sections in group 1 and group 2 were subjected to the pH cycling regime as reported previously [Fig. 1] [35]. Before the emersion cycle, the enamel sections were stored at minus 4 °C in artificial saliva for 24 h to control the immediate acid production by the bacteria. All the sections were given the following treatment to mimic the natural pH cycle in a day. The pH of the suspension was checked before starting the pH cycle. The specimens were immersed for in 40 mL of respective solution [probiotic solution for group 1 and demineralizing agent (0.1 M Lactic acid) for group 2] under agitation for 6 h at 37 degrees Celsius. After each immersion cycle, the specimens were washed with distilled water for one minute and maintained in 10 mL of artificial saliva at 37 °C for 16 h until the next immersion cycle, and this was repeated 6 times. The solution in all the groups was replaced before each immersion cycle [35]. Thymol was added to each of the solutions to avoid fungal growth.

The Vickers microhardness, surface roughness, surface morphology, and elemental composition of enamel [Carbon [C], Oxygen [O], Sodium [Na], Hydrogen [H], Magnesium [Mg], Phosphorus [P], Fluoride [F], Chlorine [Cl], and Calcium [Ca]] were checked before and after the erosive challenge. The surfaces of enamel were checked under SEM for the presence of any changes in the surface morphology such as roughness, fine lines, scratches, gouges, cracks, pitting, and any remnants of debris. The intergroup and intragroup comparison for surface roughness, microhardness, and changes in the elemental composition was done by Wilcoxon signed rank test and Mann–Whitney U test followed by Bonferroni correction. *P* < 0.05 was considered to be significant.

The changes in the pH in the probiotic solution were checked at baseline and after 2, 4, 6, 12, and 24 h by mixing the probiotic sachets in the Fermentation Minimal Medium [FMN] as described previously [6]. FMM helps the bacteria survive and helps calculate the amount of acid produced and the change in the pH over time. FMM was formulated by mixing 50 mM KCl, 5 mM NaCl, 2 mM MgSO_4_, 2 mM MnCl_2_, 8 mM [NH_4_]_2_SO_4_, 1.5 µM thiamine, and 8 µM niacin at pH 7.0 as described elsewhere [7]. Then, 1 mL of the bacterial suspension in FMM was introduced into 21 different aliquots. The bacteria were incubated at 37ºC for 20 min in a water bath without agitation. This was done to deplete the endogenous carbohydrate reserves**.** The pH of the probiotic solution and demineralizing solution was subsequently recorded by pipetting the solution from each well at baseline, 1, 2, 4, 6, 12, and 24 h by using a pH meter [Eutech pH Meter PH 700, LabFriend, India]. The mean descriptive of all the baseline and post-pH cycle regime measurements were done.

## Results

This study is only providing evidence that the amount of acid produced by probiotic bacteria results in enamel defects that are not significantly different from that, produced by 0.1 M Lactate (Table 1). A steady decrease in the pH of the probiotic solution in FMM media was noted, thereby confirming the increase in the acidity of the probiotic solution with time. The mean pH at 0, 2, 4, 6, 8, 12, and 24 h dropped from 5.78 to 4.74, 4.48, 3.93, 3.57, 3.19, and 3.06, respectively. The mean pH in the demineralizing group was 4.86 at baseline which dropped to 4.92, 4.42, 4.27, 4.27, and 4.14, and 3.95 at 2, 4, 6, 8, 12, and 24 h, respectively. We noted the rise in the lactic acid produced in the probiotic group with the mean lactic acid concentration at baseline, 2, 4, 6, 8, 12, and 24 h was found to be 0.012 mg/ml, 0.020 mg/ml, 0.128 mg/ml, 0.142 mg/ml, 0.144 mg/ml, and 0.149 mg/ml.

**Table 1:** Intergroup comparison between probiotic solution and demineralizing agent (0.1 Lactic acid) using Mann–Whitney *U* test (independent samples) and Wilcoxon rank test

Test statistics	CW	CA	OW	OA	FW	FA
Mann–Whitney U	33.50	31.00	49.00	39.50	33.00	33.00
Wilcoxon W	88.50	86.00	104.00	94.50	88.00	88.00
Z	− 1.25	− 1.44	− 0.076	− 0.796	− 1.46	− 1.46
Asymp. Sig. (2-tailed)	0.211	0.150	0.940	0.426	0.143	0.143
Exact Sig. [2*(1-tailed Sig.)]	0.218^b^	0.165^b^	0.971^b^	0.436^b^	0.218^b^	0.218^b^

Test statistics	NW	NA	MgW	MgA	AlW	AlA
Mann–Whitney U	45.00	45.00	50.00	50.00	32.50	46.00
Wilcoxon W	100.00	100.00	105.00	105.00	87.50	101.00
Z	− 0.61	− 1.00	0.00	0.00	− 1.535	− 0.40
Asymp. Sig. (2-tailed)	0.54	0.31	1.000	1.000	0.125	0.689
Exact Sig. [2*(1-tailed Sig.)]	0.739^b^	0.739^b^	1.000^b^	1.000^b^	0.190^b^	0.796^b^

Test statistics	SiW	SiA	PW	PA	ClW	ClA
Mann–Whitney U	40.00	45.00	33.50	33.00	49.00	45.00
Wilcoxon W	95.00	100.00	88.50	88.00	104.00	100.00
Z	− 1.453	− 1.000	− 1.261	− 1.306	− 0.108	− 1.000
Asymp. Sig. (2-tailed)	0.146	0.317	0.207	0.192	0.914	0.317
Exact Sig. [2*(1-tailed Sig.)]	0.481^b^	0.739^b^	0.218^b^	0.218^b^	0.971^b^	0.739^b^

Test statistics^a^	KW	KA	CaW	CaA	NW	NA	SW	SA
Mann–Whitney U	40.00	50.000	33.00	33.00	38.50	28.50	50.00	50.00
Wilcoxon W	95.00	105.00	88.00	88.00	93.50	83.50	105.00	105.00
Z	− 1.45	0.00	− 1.288	− 1.291	− 0.878	− 1.631	0.000	0.000
Asymp. Sig. (2-tailed)	0.146	1.00	0.198	0.197	0.380	0.103	1.000	1.000
Exact Sig. [2*(1-tailed Sig.)]	0.481^b^	1.000^b^	0.218^b^	0.218^b^	0.393^b^	0.105^b^	1.000^b^	1.000^b^

^a^Grouping Variable: 1

^b^Not corrected for ties.

The effect of probiotics on the surface microhardness, microtopography, and change in the elemental composition of enamel when mixed with artificial saliva was assessed and is mentioned in Table 2 and Table 3. At baseline, the mean surface roughness for enamel surface exposed to both group 1 (probiotics) and group 2 (0.1 M lactic acids) was 3.3 ± 0.8 and 3.2 ± 0.6, respectively. Post-exposure, the mean surface roughness in group 1 and group 2 increased to 3.8 ± 0.7 [*P* = 0.005] and 4.00 ± 0.6 [*P* = 0.009]. The intragroup comparison done using the Wilcoxon rank test for group 1 and group 2 showed a significant increase in surface roughness before and after exposure [N-10; Group 1: Z: − 2.803b, Asymp. Sig. [2-tailed] *P* = 0.05 and Group 2: Z:2.599b, *P* = 0.0097]]. Upon intergroup comparison between group 1 and group 2, no significant difference was noted for the surface roughness between the two groups [Z = [9]− 0.708; Exact Sig. [2*[1-tailed Sig.]] = 0.479]. The mean Vickers’ microhardness in group 1 and group 2 at baseline was 227.10 ± 42.54 VHN and 211.60 ± 51.78 VHN, respectively. Post-exposure, the mean microhardness decreased in both the groups [group 1 = 165.60 ± 36.35 [*P* = 0.005]; group 2 = 172.10 ± 29.87 [*P* = 0.009]. Upon comparison between pre- and post-exposure between group 1 and group 2. The probiotic group showed a significant decrease in the microhardness [Median [pre-post]: 65; Z: − 2.80b, *P* value = 0.005]. Upon intergroup comparison between group 1 and group 2, no significant difference was noted for the surface roughness between the two groups [Z = − 0.983; Exact Sig. [2*[1-tailed Sig.]] = 0.353b].

**Table 2:** Descriptive summary of the mean surface roughness, microhardness, and elemental composition of enamel sections for Calcium (Ca), Carbon (C), Oxygen (O), Fluoride (F), Sodium (Na), Magnesium (Mg), Aluminium (Al), Silicon (Si), Phosphorus (P), Potassium (K), before and after exposure to probiotic solution

Group 1 probiotic solution	Samples	Baseline (before exposure)						
Outcomes	*N*	Mean	Std. deviation	Minimum	Maximum	Percentiles		
						25th	50th	75th
CW	10	17.50	14.52	0	49	9.00	14.00	22.00
CA	10	26.30	17.80	1	61	16.00	23.00	33.50
OW	10	38.30	5.35	27	45	35.75	40.00	42.00
OA	10	48.10	10.09	29	57	44.25	51.50	54.25
FW	10	0.60	0.69	0	2	0.00	0.50	1.00
FA	10	0.60	0.69	0	2	0.00	0.50	1.00
NaW	10	0.40	0.51	0	1	0.00	0.00	1.00
NaA	10	0.20	0.42	0	1	0.00	0.00	0.25
MgW	10	0.00	0.00	0	0	0.00	0.00	0.00
MgA	10	0.00	0.00	0	0	0.00	0.00	0.00
AlW	10	0.40	0.51	0	1	0.00	0.00	1.00
AlA	10	0.40	0.51	0	1	0.00	0.00	1.00
SiW	10	0.00	0.00	0	0	0.00	0.00	0.00
SiA	10	0.00	.00	0	0	0.00	0.00	0.00
PW	10	14.80	3.70	7	21	12.75	15.50	17.00
PA	10	9.70	3.56	3	16	7.50	10.00	12.00
ClW	10	0.50	0.52	0	1	0.00	0.50	1.00
ClA	10	0.10	0.31	0	1	0.00	0.00	0.00
KW	10	0.00	0.00	0	0	0.00	0.00	0.00
KA	10	0.00	0.000	0	0	0.00	0.00	0.00
CaW	10	26.80	8.12	9	40	23.50	27.50	31.00
CaA	10	13.70	5.73	3	25	11.25	13.50	16.50
NW	10	0.20	0.422	0	1	0.00	0.00	0.25
NA	10	0.20	0.422	0	1	0.00	0.00	0.25
SW	10	0.00	0.00	0	0	0.00	0.00	0.00
SA	10	0.00	0.00	0	0	0.00	0.00	0.00
PR	10	3.30	0.823	2	5	3.00	3.00	4.00
VHN	10	227.10	42.540	158	301	203.00	225.50	254.75

Group 1 probiotic solution	Post-exposure							*Z* value	*P* value	Adjusted *P*-value based on Bonferroni corrections
Outcomes	Mean	Std. Deviation	Minimum	Maximum	Percentiles			Pre-Post		
					25th	50th	75th			
CW	35.80	16.92	11	60	22.50	34.50	54.50	− 2.43^b^	0.015	0.045
CA	46.50	16.46	18	68	33.50	49.00	63.00	− 2.34^b^	0.019	0.057
OW	31.60	10.81	17	44	21.25	33.00	43.25	− 1.43^c^	0.153	0.459
OA	33.80	14.55	15	53	19.00	35.00	47.25	− 1.98^c^	0.047	0.141
FW	0.20	0.42	0	1	0.00	0.00	0.25	− 1.41^c^	0.157	0.471
FA	0.20	0.42	0	1	0.00	0.00	0.25	− 1.41^b^	0.157	0.471
NaW	0.40	0.51	0	1	0.00	0.00	1.00	0.00^b^	1.00	3.00
NaA	0.30	0.48	0	1	0.00	0.00	1.00	− 0.44^c^	0.655	1.965
MgW	0.00	0.00	0	0	0.00	0.00	0.00	0.00^c^	1.000	3.00
MgA	0.00	0.00	0	0	0.00	0.00	0.00	0.00^b^	1.000	3.00
AlW	0.60	0.96	0	3	0.00	0.00	1.00	− 0.71^b^	0.480	1.44
AlA	0.30	0.67	0	2	0.00	0.00	0.25	− 577^c^	0.564	1.692
SiW	0.20	0.42	0	1	0.00	0.00	0.25	− 1.41^c^	0.157	0.471
SiA	0.10	0.31	0	1	0.00	0.00	0.00	− 1.00^b^	0.317	0.951
PW	8.30	5.47	0	14	1.50	10.50	11.75	− 2.81^b^	0.005	0.015
PA	4.80	3.36	0	9	0.75	6.00	6.75	− 2.67^c^	0.008	0.024
ClW	0.70	0.67	0	2	0.00	1.00	1.00	− 707^c^	0.480	1.44
ClA	0.10	0.31	0	1	0.00	0.00	0.00	0.00^c^	1.000	3.00
KW	0.30	0.48	0	1	0.00	0.00	1.00	− 1.73^b^	0.083	0.249
KA	0.00	0.000	0	0	0.00	0.00	0.00	0.00^b^	1.000	3.00
CaW	14.80	9.18	1	27	4.75	17.00	21.25	− 2.81^c^	0.005	0.015
CaA	6.40	4.50	0	13	1.50	7.00	9.00	− 2.81^c^	0.005	0.015
NW	6.30	6.55	0	17	0.00	3.50	12.50	− 2.37^b^	0.018	0.054
NA	6.80	6.74	0	18	0.00	5.00	13.25	− 2.37^b^	0.018	0.054
SW	0.00	0.00	0	0	0.00	0.00	0.00	0.00^c^	1.000	3.00
SA	0.00	0.00	0	0	0.00	0.00	0.00	0.00^c^	1.000	3.00
PR	3.80	0.78	3	5	3.00	4.00	4.25	− 2.80^b^	0.005	0.015
VHN	165.60	36.35	114	230	140.50	160.50	202.50	-2.80^b^	0.005	0.015

*CW* Caron Weight %, *CA* Caron Atomic %, *OW* Oxygen Weight %, *OA* Oxygen Atomic %, *FW* Fluoride Weight %, *FA* Fluoride Atomic %, *NaW* Sodium Weight %, *NaA* Sodium Atomic %, *MgW* Magnesium Weight %, *MgA* Magnesium Atomic %, *AlW* Aluminium Weight %, *AlA* Aluminium Atomic %, *SiW* Silicon Weight %, *SiA* Silicon Atomic %, *PW* Phosphorus Weight %, *PA* Phosphorus Atomic %, *ClW* Chlorine Weight %, *ClA* Chlorine Atomic %, *KW* Potassium Weight %, *KA* Potassium Atomic *CaW* Calcium Weight %, *CaA* Calcium Atomic, *NW* Nitrogen Weight %, *NA* Nitrogen Atomic %, *SW* Sulphur Weight %, *SA* Sulphur Atomic %, *PR* Profilometer VHN Vicker's Hardness

^a^Wilcoxon signed rank test

^b^Based on positive ranks

^c^Based on negative ranks

^d^The sum of negative ranks equals the sum of positive ranks

e. *P* < 0.05 is considered statistically significant

**Table 3:** Descriptive summary of the mean surface roughness, microhardness and elemental composition of enamel sections for Calcium (Ca), Carbon (C), Oxygen (O), Fluoride (F), Sodium (Na), Magnesium (Mg), Aluminium (Al), Silicon (Si), Phosphorus (P), Potassium (K), before and after exposure to a demineralizing agent (0.1 M lactic acid)

Group 2 standard demineralizing agent	Samples	(Before exposure)						
Outcomes	*N*	Mean	Std. Deviation	Minimum	Maximum	Percentiles		
						25th	50th	75th
CW	10	18.30	14.46	2	44	3.75	16.50	28.25
CA	10	26.90	18.84	4	58	6.50	26.50	41.25
OW	10	37.00	4.98	29	43	32.50	38.00	41.50
OA	10	46.00	11.46	29	61	34.50	47.00	56.50
FW	10	0.60	0.51	0	1	0.00	1.00	1.00
FA	10	0.70	0.48	0	1	0.00	1.00	1.00
NaW	10	0.20	0.42	0	1	0.00	0.00	0.25
NaA	10	0.20	0.42	0	1	0.00	0.00	0.25
MgW	10	0.00	0.00	0	0	0.00	0.00	0.00
MgA	10	0.00	0.00	0	0	0.00	0.00	0.00
AlW	10	0.60	0.69	0	2	0.00	0.50	1.00
AlA	10	0.20	0.42	0	1	0.00	0.00	0.25
SiW	10	0.00	0.00	0	0	0.00	0.00	0.00
SiA	10	0.00	0.00	0	0	0.00	0.00	0.00
PW	10	14.30	4.19	9	20	9.75	15.00	19.00
PA	10	9.50	3.92	5	15	5.00	10.00	14.00
ClW	10	0.40	0.51	0	1	0.00	0.00	1.00
ClA	10	0.00	0.00	0	0	0.00	0.00	0.00
KW	10	0.00	0.00	0	0	0.00	0.00	0.00
KA	10	0.00	0.00	0	0	0.00	0.00	0.00
CaW	10	26.00	8.35	15	38	16.00	28.00	34.00
CaA	10	13.20	5.78	6	22	6.75	14.00	19.00
NW	10	2.10	6.29	0	20	0.00	0.00	0.25
NA	10	2.60	7.54	0	24	0.00	0.00	0.50
SW	10	0.00	0.00	0	0	0.00	0.00	0.00
SA	10	0.00	0.00	0	0	0.00	0.00	0.00
PR	10	3.20	0.63	2	4	3.00	3.00	4.00
VHN	10	211.60	51.78	135	298	168.75	220.00	249.00

Group 2 standard demineralizing agent	Post-exposure							*Z* value	*P* value	Adjusted *P* values based on bonferroni corrections
Outcomes	Mean	Std. deviation	Minimum	Maximum	Percentiles			Pre-Post		
					25th	50th	75th			
CW	26.50	21.29	2	56	3.00	25.50	44.75	− 1.820^b^	0.069	0.207
CA	34.80	25.14	3	64	5.75	36.50	59.50	− 1.660^b^	0.097	0.291
OW	30.20	11.41	11	43	21.00	31.50	41.25	− 2.103^c^	0.035	0.105
OA	34.30	16.13	11	54	19.00	37.50	48.75	− 2.805^c^	0.005	0.015
FW	0.80	0.92	0	2	0.00	0.50	2.00	− 0.707^b^	0.480	1.44
FA	0.80	0.92	0	2	0.00	0.50	2.00	− 0.447^b^	0.655	1.96
NaW	0.50	0.57	0	1	0.00	0.50	1.00	− 1.732^b^	0.083	0.249
NaA	0.50	0.57	0	1	0.00	0.50	1.00	− 1.732^c^	0.083	0.249
MgW	0.00	0.00	0	0	0.00	0.00	0.00	0.000^c^	1.000	3.00
MgA	0.00	0.00	0	0	0.00	0.00	0.00	0.000^b^	1.000	3.00
AlW	0.00	0.00	0	0	0.00	0.00	0.00	− 2.121^b^	0.034	0.102
AlA	0.00	0.00	0	0	0.00	0.00	0.00	− 1.414^c^	0.157	0.471
SiW	0.00	0.00	0	0	0.00	0.00	0.00	0.000^c^	1.000	3.00
SiA	0.00	0.00	0	0	0.00	0.00	0.00	0.000^b^	1.000	3.00
PW	10.60	4.94	2	17	7.50	11.00	15.00	− 2.611^b^	0.009	0.027
PA	6.50	3.68	1	12	4.00	6.50	10.00	− 2.717^c^	0.007	0.021
ClW	0.40	0.516	0	1	0.00	0.00	1.00	0.000^c^	1.000	3.00
ClA	0.10	0.316	0	1	0.00	0.00	0.00	− 1.000^b^	0.317	0.951
KW	0.00	0.000	0	0	0.00	0.00	0.00	− 1.732^b^	0.083	0.249
KA	0.00	0.000	0	0	0.00	0.00	0.00	0.000^b^	1.000	3.00
CaW	19.50	9.41	3	32	13.00	22.00	25.25	− 2.403^c^	0.016	0.048
CaA	9.00	5.14	1	17	4.75	9.50	13.00	− 2.609^c^	0.009	0.027
NW	11.10	2.99	4	15	9.75	12.00	13.00	− 2.616^b^	0.009	0.027
NA	13.70	4.49	4	20	11.75	13.50	18.00	− 2.603^b^	0.009	0.027
SW	0.00	0.000	0	0	.00	.00	0.00	0.000^c^	1.000	3.00
SA	0.00	0.000	0	0	.00	.00	0.00	0.000^c^	1.000	3.00
PR	4.00	0.667	3	5	3.75	4.00	4.25	− 2.599^b^	0.009	0.027
VHN	172.10	29.872	126	214	144.25	181.50	195.50	− 2.599^b^	0.009	0.027

*CW* Caron Weight %, *CA* Caron Atomic %, *OW* Oxygen Weight %, *OA* Oxygen Atomic %, *FW* Fluoride Weight %, *FA* Fluoride Atomic %, *NaW* Sodium Weight %, *NaA* Sodium Atomic %, *MgW* Magnesium Weight %, *MgA* Magnesium Atomic %, *AlW* Aluminium Weight %, *AlA* Aluminium Atomic %, *SiW* Silicon Weight %, *SiA* Silicon Atomic %, *PW* Phosphorus Weight %, *PA* Phosphorus Atomic %, *ClW* Chlorine Weight %, *ClA* Chlorine Atomic %, *KW* Potassium Weight %, *KA* Potassium Atomic *CaW* Calcium Weight %, *CaA* Calcium Atomic, *NW* Nitrogen Weight %, *NA* Nitrogen Atomic %, *SW* Sulphur Weight %, *SA* Sulphur Atomic %, *PR* Profilometer *VHN* Vicker's Hardness

^a^Wilcoxon signed rank test

^b^Based on positive ranks

^c^Based on negative ranks

^d^The sum of negative ranks equals the sum of positive ranks

e. *P* < 0.05 is considered statistically significant

**Fig. 2:**
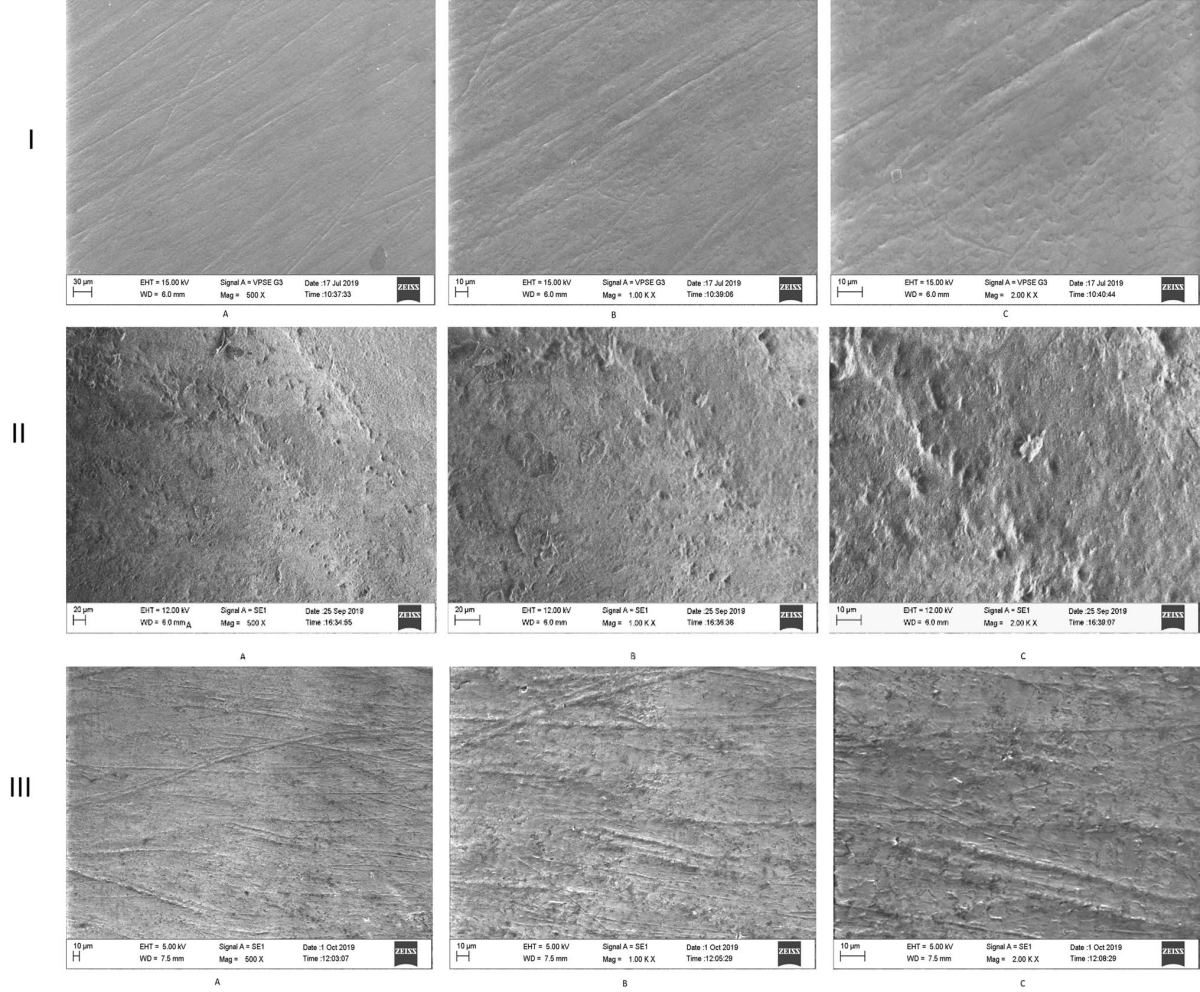
Comparison of the surface topography of enamel specimen observed under Scanning Electron Microscope [SEM] [EVO MA 18 with Oxford EDS] [A, B, and C are images captured at 500x, 1000 ×, and 2000 ×, respectively]: I. Surface topography of enamel specimen before any acid challenge; II. Surface topography of enamel specimen after exposure to oral probiotic suspension Darolac [Aristo Pharmaceuticals Pvt Ltd]; III. Surface topography of enamel specimen after exposure to Standard Demineralizing agent (0.1 M Lactic acid)

The surface morphology as measured by the SEM showed an increase in the number of striations, scratch marks, and pitting on the enamel surface. An altered arrangement of the enamel prisms was noted for both the probiotic group and demineralizing group; however, the sections with demineralizing agent showed more pitting and roughness in the enamel surface morphology. At increased magnification, loss of enamel prism core with inter-prismatic enamel protruding along with damaged and fragmented enamel was seen in both groups [Fig. 2]. A honeycombed pattern can be appreciated in the demineralizing group [Fig. 2 IIIC]. Erosion was appreciated with loss of the prism core and prism sheath with some areas showing the formation of pits, pores, and micro-erosion in higher magnification in both the probiotic and demineralizing groups. Globular rod-shaped crystals were also seen in some of the eroded areas [Fig. 2 IIC].

The changes in the elemental composition of C, N, S, Ca, P, K, Na, Al, Si, Cl, and Mg were noted using SEM/EDX at 500X, 1000X, and 2000X in both groups. A decrease in the atomic/weight % for Ca, P, Fl, Al, and O and an increase in weight/atomic % for C, N, and Na were noted compared to the baseline in the probiotic solution [Table 2]. The intragroup comparison between the pre- and post-exposure showed that C, Ca, and P showed the maximum change upon exposure to the probiotic supplements. The weight percentage for Ca [Median-pre: 27.50; post:17.00; Z: − 2.81; *P* value: 0.005, adjusted *p* value: 0.015] and *P* [Median-pre:15.50; post: 10.50; Z: 2.81b; *P* value: 0.005; adjusted *p* value: 0.015] after exposure to the probiotic solution was significantly reduced as compared to baseline [Tables1–3, and Supplementary Tables 1 and 2]. The maximum difference in the median value was noted with respect to C [20]; O [− 7]; Ca [− 10.5]; and P [− 5]. The intergroup comparison with the Wilcoxon rank test and Mann–Whitney *U* test showed that the mean rank for probiotic solution compared to demineralizing agent was higher for atomic percentage for C and Si and weight percentage for C, Al, and K. However, there was no statistically significant difference between the two groups for any of the elements measured. Similar mean ranks were noted for the atomic percentage of S, C, K, Cl, Si, Al, Mg, and N and the weight percentage of C, Cl, Mg, N, and O [Supplementary Table 2, Fig. 2].

## Discussion

Oral microbiome is a complex, diverse, and highly dynamic ecosystem [36]. It harbours more than 700 microbial species, which are either beneficial or pathogenic in nature [37]. Every microbial species in the oral ecosystem is affected by other members of the microbial community. The growth and survival of bacteria in the biofilm are also influenced by many patient-related and environmental factors (extrinsic or intrinsic) [36–38]. Among all the environmental factors, our dietary choices play a crucial role in modulating the oral microbiome. [39]

The acidity, pH, and composition of our diet influence the amount, growth, survival, and diversity of bacteria in the oral biofilm [39]. For example, increased consumption of acidic and sugary carbonated drinks or beverages increase the acidity of the saliva, favouring the growth of many acid-tolerant bacteria in the community. This leads to loss of bacterial diversity, dysbiosis, and onset of disease. The increased acidity drops the pH of the saliva and causes essential elements like Calcium and Phosphorus to be released from the tooth surface. This initiates the process of demineralization and increase the risk of tooth decay [33]. Similar to carbonated acidic drinks, probiotic formulations can also add to the existing acidity of the oral cavity. This has been previously explained by the “ecological plaque hypothesis” by Marsh et al. (2003), [38] who stated that the demineralization of enamel need not have a specific microbial aetiology and any species, conditions, or habit (consumption of acidic beverages or drinks), which can increase the acidic load in the oral environment, should be considered as a risk factor for enamel demineralization and caries [38]. Recently in 2020, the integrated plaque hypothesis also stated that shift in the ecological balance with increase in the acidity or alkalinity of the oral environment increases the risk of the oral disease. When the pH of the oral cavity is reduced and the mineral balance tilts towards demineralization, the cariogenic processes may progress and this result in the development of a clinically visible root caries lesion [39]. The acidic environment promotes the introduction of more acidogenic bacteria, which are more capable of lowering the environmental pH [40]. Therefore, to maintain or restore the dynamic stability of the oral health, it is necessary to control the drivers that maintain the pH of the oral cavity. Considering these concepts, the role of probiotics formulations containing live acidogenic bacteria, that produce large amount acid, on enamel surface is deemed crucial.

Previous studies have confirmed that probiotic bacteria can reduce the pH of the saliva and adds to existing acidity of the oral biofilm [41–45]. The probiotics can even integrate with other members of the oral biofilm and favours the growth of other acidogenic bacteria such as *Lactobacillus acidophilus, Bifidobacterium dentium, Bifidobacterium longum, and S. mutans* [43–46]. Few studies have also found that *Lactobacilli* increase the levels of *Streptococci* species in the dental plaque and increase the risk of caries by increasing the formation of extracellular polysaccharide, that is used for biofilm formation [47, 48]. A study by Haukoija et al. noted that probiotic bacteria, especially *Lactobacillus casei* and *Lactobacillus acidophilus,* can induce caries, even in the absence of *Streptococcus mutans.* The ability of this probiotic bacteria to produce lactic acid is increased in the presence of supragingival plaque [6, 13, 47]. Our study also noted a fall in the pH and demineralization of enamel section when immersed in probiotic solutions. We did not find any difference in the microhardness, surface roughness, and elemental composition of enamel when samples were immersed in probiotic solution or 0.1 M Lactate. However, the loss of essential elements proved that some amount of demineralization has occurred. These results can also be supported by studies by Ferrer et al. (2020), Singh and Dole (2016), and Angarita et al. (2020) where probiotic consumption was found to increase the “superficial loss of calcium and phosphorous from the enamel surface” [22, 27, 42]. However, there are few studies that negate this as the ability of probiotic drinks to lower the pH of the saliva was minimal and no change in pH was noted upon probiotic consumption owing to the buffering capacity of saliva and high Calcium content in the probiotic drink [48–50].

However, one should note that although probiotics may lead to leaching of essential elements from the enamel, these changes are insignificant to be visible clinically. The buffering action of saliva and onset of the cycle of remineralization may protect the enamel and restore the lost minerals. However, if probiotics integrate with oral biofilm and survive in the oral cavity for long time and there is regular consumption of probiotics, the effect would be significant [51]. Our study proved for the first time that probiotic can alter the pH and affect the microhardness, surface roughness, and essential elements like Calcium and Phosphorus to leach from the enamel. But, there are some limitations in our study; we have not calculated a shift in the bacterial composition of the biofilm in the direction of more acidic species. As we have not done any further research into this shift, we would not be able to comment on this [46]. Thus, future studies should explore and confirm the long-term effects of probiotics on the tooth surface. Although our study has tried to generate preliminary evidence on the effect of probiotics on the microhardness, surface topography, and elemental composition of enamel, one should note that this study is done in in vitro settings. Hence, future studies should explore the effect of the individual probiotic bacteria on the enamel and dentin via conducting patient-based clinical studies. Studies can also compare the effect of probiotics based on the nature and mode of delivery of probiotic formulation, interaction of probiotic bacteria with other microorganisms in the oral biofilm, other dietary choices, and oral hygiene of the patients. Future studies can assess if probiotics have any role in development of non-carious cervical lesions, root lesions, erosion and demineralized enamel, and dentinal hypersenstivity.

## Conclusion

Probiotic bacteria are acidogenic in nature. The emersion of enamel in probiotic solution decrease the pH and increase the Lactic acid concentration in the solution. This affects the integrity of the enamel as it reduces the microhardness, increases the surface roughness, and alters elemental composition of enamel. Exposure to probiotics can cause leaching of essential elements like Calcium and Phosphorus from the enamel, and these results are comparable to 0.1 M lactic acid. Thus, we conclude that exposure to probiotics increase the risk of enamel demineralization; however, these results should be confirmed by performing similar studies in clinical settings.

## Supplementary Information

Below is the link to the electronic supplementary material.

Supplementary file1 (DOCX 33 KB)

## Data Availability

Data is available with the corresponding author and can be shared on request via an email to aditi.chopra@manipal.edu.
