# Supplementary material for: Can acid produced from probiotic bacteria alter the surface roughness, microhardness, and elemental composition of enamel? An in vitro study
Source: Odontology. 2023 Mar 30;111(4):929–41. doi: 10.1007/s10266-023-00804-1 (PMC10492682; doi:10.1007/s10266-023-00804-1)
Supplement: Supplementary file 1 — Supplementary file1 (DOCX 33 KB) [file 10266_2023_804_MOESM1_ESM.docx]

**Supplementary Table 1: Comparison of the changes in the elemental composition, surface roughness, microhardness of enamels before and after exposure to probiotic solution (group 1) and standard demineralizing agent (group 2) as analysed by** **Wilcoxon Signed Ranks Test.**

| **Ranks** | | | | | |
| --- | --- | --- | --- | --- | --- |
| Probiotic group (group 1) | | | N | Mean Rank | Sum of Ranks |
| 1 | CBW - CPW | Negative Ranks | 8^a^ | 5.38 | 43.00 |
|  |  | Positive Ranks | 1^b^ | 2.00 | 2.00 |
|  |  | Ties | 1^c^ |  |  |
|  |  | Total | 10 |  |  |
|  | CBA - CPA | Negative Ranks | 8^d^ | 6.31 | 50.50 |
|  |  | Positive Ranks | 2^e^ | 2.25 | 4.50 |
|  |  | Ties | 0^f^ |  |  |
|  |  | Total | 10 |  |  |
|  | OBW - OPW | Negative Ranks | 4^g^ | 3.38 | 13.50 |
|  |  | Positive Ranks | 6^h^ | 6.92 | 41.50 |
|  |  | Ties | 0^i^ |  |  |
|  |  | Total | 10 |  |  |
|  | OBA - OPA | Negative Ranks | 2^j^ | 4.00 | 8.00 |
|  |  | Positive Ranks | 8^k^ | 5.88 | 47.00 |
|  |  | Ties | 0^l^ |  |  |
|  |  | Total | 10 |  |  |
|  | FBW - FPW | Negative Ranks | 1^m^ | 2.50 | 2.50 |
|  |  | Positive Ranks | 4^n^ | 3.13 | 12.50 |
|  |  | Ties | 5^o^ |  |  |
|  |  | Total | 10 |  |  |
|  | FBA - FPA | Negative Ranks | 1^p^ | 2.50 | 2.50 |
|  |  | Positive Ranks | 4^q^ | 3.13 | 12.50 |
|  |  | Ties | 5^r^ |  |  |
|  |  | Total | 10 |  |  |
|  | NaBW - NaPW | Negative Ranks | 3^s^ | 3.50 | 10.50 |
|  |  | Positive Ranks | 3^t^ | 3.50 | 10.50 |
|  |  | Ties | 4^u^ |  |  |
|  |  | Total | 10 |  |  |
|  | NaBA - NaPA | Negative Ranks | 3^v^ | 3.00 | 9.00 |
|  |  | Positive Ranks | 2^w^ | 3.00 | 6.00 |
|  |  | Ties | 5^x^ |  |  |
|  |  | Total | 10 |  |  |
|  | MgBW - MgPW | Negative Ranks | 0^y^ | .00 | .00 |
|  |  | Positive Ranks | 0^z^ | .00 | .00 |
|  |  | Ties | 10^aa^ |  |  |
|  |  | Total | 10 |  |  |

| 1 | MgBA - MgPA | Negative Ranks | 0^a^ | .00 | .00 |
| --- | --- | --- | --- | --- | --- |
|  |  | Positive Ranks | 0^b^ | .00 | .00 |
|  |  | Ties | 10^c^ |  |  |
|  |  | Total | 10 |  |  |
|  | AlBW - AlPW | Negative Ranks | 3^d^ | 3.33 | 10.00 |
|  |  | Positive Ranks | 2^e^ | 2.50 | 5.00 |
|  |  | Ties | 5^f^ |  |  |
|  |  | Total | 10 |  |  |
|  | AlBA - AlPA | Negative Ranks | 1^g^ | 2.00 | 2.00 |
|  |  | Positive Ranks | 2^h^ | 2.00 | 4.00 |
|  |  | Ties | 7^i^ |  |  |
|  |  | Total | 10 |  |  |
|  | SiBW - SiPW | Negative Ranks | 2^j^ | 1.50 | 3.00 |
|  |  | Positive Ranks | 0^k^ | .00 | .00 |
|  |  | Ties | 8^l^ |  |  |
|  |  | Total | 10 |  |  |
|  | SiBA - SiPA | Negative Ranks | 1^m^ | 1.00 | 1.00 |
|  |  | Positive Ranks | 0^n^ | .00 | .00 |
|  |  | Ties | 9^o^ |  |  |
|  |  | Total | 10 |  |  |
|  | PBW - PPW | Negative Ranks | 0^p^ | .00 | .00 |
|  |  | Positive Ranks | 10^q^ | 5.50 | 55.00 |
|  |  | Ties | 0^r^ |  |  |
|  |  | Total | 10 |  |  |
|  | PBA - PPA | Negative Ranks | 0^s^ | .00 | .00 |
|  |  | Positive Ranks | 9^t^ | 5.00 | 45.00 |
|  |  | Ties | 1^u^ |  |  |
|  |  | Total | 10 |  |  |
|  | ClBW - ClPW | Negative Ranks | 3^v^ | 3.33 | 10.00 |
|  |  | Positive Ranks | 2^w^ | 2.50 | 5.00 |
|  |  | Ties | 5^x^ |  |  |
|  |  | Total | 10 |  |  |
|  | ClBA - ClPA | Negative Ranks | 1^y^ | 1.50 | 1.50 |
|  |  | Positive Ranks | 1^z^ | 1.50 | 1.50 |
|  |  | Ties | 8^aa^ |  |  |
|  |  | Total | 10 |  |  |

| 1 | KBW - KPW | Negative Ranks | 3^a^ | 2.00 | 6.00 |
| --- | --- | --- | --- | --- | --- |
|  |  | Positive Ranks | 0^b^ | .00 | .00 |
|  |  | Ties | 7^c^ |  |  |
|  |  | Total | 10 |  |  |
|  | KBA - KPA | Negative Ranks | 0^d^ | .00 | .00 |
|  |  | Positive Ranks | 0^e^ | .00 | .00 |
|  |  | Ties | 10^f^ |  |  |
|  |  | Total | 10 |  |  |
|  | CaBW- CaPW | Negative Ranks | 0^g^ | .00 | .00 |
|  |  | Positive Ranks | 10^h^ | 5.50 | 55.00 |
|  |  | Ties | 0^i^ |  |  |
|  |  | Total | 10 |  |  |
|  | CaBA - CaPA | Negative Ranks | 0^j^ | .00 | .00 |
|  |  | Positive Ranks | 10^k^ | 5.50 | 55.00 |
|  |  | Ties | 0^l^ |  |  |
|  |  | Total | 10 |  |  |
|  | NBW - NPW | Negative Ranks | 7^m^ | 4.00 | 28.00 |
|  |  | Positive Ranks | 0^n^ | .00 | .00 |
|  |  | Ties | 3^o^ |  |  |
|  |  | Total | 10 |  |  |
|  | NBA - NPA | Negative Ranks | 7^p^ | 4.00 | 28.00 |
|  |  | Positive Ranks | 0^q^ | .00 | .00 |
|  |  | Ties | 3^r^ |  |  |
|  |  | Total | 10 |  |  |
|  | SBW - SPW | Negative Ranks | 0^s^ | .00 | .00 |
|  |  | Positive Ranks | 0^t^ | .00 | .00 |
|  |  | Ties | 10^u^ |  |  |
|  |  | Total | 10 |  |  |
|  | SBA - SPA | Negative Ranks | 0^v^ | .00 | .00 |
|  |  | Positive Ranks | 0^w^ | .00 | .00 |
|  |  | Ties | 10^x^ |  |  |
|  |  | Total | 10 |  |  |
|  | PRB - PRP | Negative Ranks | 6^y^ | 4.00 | 24.00 |
|  |  | Positive Ranks | 1^z^ | 4.00 | 4.00 |
|  |  | Ties | 3^aa^ |  |  |
|  |  | Total | 10 |  |  |
|  | VHNB - VHNP | Negative Ranks | 0^a^ | .00 | .00 |
|  |  | Positive Ranks | 10^b^ | 5.50 | 55.00 |
|  |  | Ties | 0^c^ |  |  |
|  |  | Total | 10 |  |  |
|  | Standard demineralizing group (Group 2) | N | Mean Rank | Sum of Ranks |  |
| Wilcoxon rank test for standard demineralizing agent | | | | | |
| 2 | CBW - CPW | Negative Ranks | 6^d^ | 5.17 | 31.00 |
|  |  | Positive Ranks | 2^e^ | 2.50 | 5.00 |
|  |  | Ties | 2^f^ |  |  |
|  |  | Total | 10 |  |  |
|  | CBA - CPA | Negative Ranks | 6^g^ | 6.08 | 36.50 |
|  |  | Positive Ranks | 3^h^ | 2.83 | 8.50 |
|  |  | Ties | 1^i^ |  |  |
|  |  | Total | 10 |  |  |
|  | OBW - OPW | Negative Ranks | 2^j^ | 1.50 | 3.00 |
|  |  | Positive Ranks | 6^k^ | 5.50 | 33.00 |
|  |  | Ties | 2^l^ |  |  |
|  |  | Total | 10 |  |  |
|  | OBA - OPA | Negative Ranks | 0^m^ | .00 | .00 |
|  |  | Positive Ranks | 10^n^ | 5.50 | 55.00 |
|  |  | Ties | 0^o^ |  |  |
|  |  | Total | 10 |  |  |
|  | FBW - FPW | Negative Ranks | 3^p^ | 3.33 | 10.00 |
|  |  | Positive Ranks | 2^q^ | 2.50 | 5.00 |
|  |  | Ties | 5^r^ |  |  |
|  |  | Total | 10 |  |  |
|  | FBA - FPA | Negative Ranks | 3^s^ | 3.00 | 9.00 |
|  |  | Positive Ranks | 2^t^ | 3.00 | 6.00 |
|  |  | Ties | 5^u^ |  |  |
|  |  | Total | 10 |  |  |
|  | NaBW - NaPW | Negative Ranks | 3^v^ | 2.00 | 6.00 |
|  |  | Positive Ranks | 0^w^ | .00 | .00 |
|  |  | Ties | 7^x^ |  |  |
|  |  | Total | 10 |  |  |
|  | NaBA - NaPA | Negative Ranks | 3^y^ | 2.00 | 6.00 |
|  |  | Positive Ranks | 0^z^ | .00 | .00 |
|  |  | Ties | 7^aa^ |  |  |
|  |  | Total | 10 |  |  |

| **Ranks** | | | | | |
| --- | --- | --- | --- | --- | --- |
| Standard demineralizing solution (group 2) | | | N | Mean Rank | Sum of Ranks |
| 2 | MgBW - MgPW | Negative Ranks | 0^a^ | .00 | .00 |
|  |  | Positive Ranks | 0^b^ | .00 | .00 |
|  |  | Ties | 10^c^ |  |  |
|  |  | Total | 10 |  |  |
|  | MgBA - MgPA | Negative Ranks | 0^d^ | .00 | .00 |
|  |  | Positive Ranks | 0^e^ | .00 | .00 |
|  |  | Ties | 10^f^ |  |  |
|  |  | Total | 10 |  |  |
|  | AlBW - AlPW | Negative Ranks | 0^g^ | .00 | .00 |
|  |  | Positive Ranks | 5^h^ | 3.00 | 15.00 |
|  |  | Ties | 5^i^ |  |  |
|  |  | Total | 10 |  |  |
|  | AlBA - AlPA | Negative Ranks | 0^j^ | .00 | .00 |
|  |  | Positive Ranks | 2^k^ | 1.50 | 3.00 |
|  |  | Ties | 8^l^ |  |  |
|  |  | Total | 10 |  |  |
|  | SiBW - SiPW | Negative Ranks | 0^m^ | .00 | .00 |
|  |  | Positive Ranks | 0^n^ | .00 | .00 |
|  |  | Ties | 10^o^ |  |  |
|  |  | Total | 10 |  |  |
|  | SiBA - SiPA | Negative Ranks | 0^p^ | .00 | .00 |
|  |  | Positive Ranks | 0^q^ | .00 | .00 |
|  |  | Ties | 10^r^ |  |  |
|  |  | Total | 10 |  |  |
|  | PBW - PPW | Negative Ranks | 1^s^ | 2.00 | 2.00 |
|  |  | Positive Ranks | 9^t^ | 5.89 | 53.00 |
|  |  | Ties | 0^u^ |  |  |
|  |  | Total | 10 |  |  |
|  | PBA - PPA | Negative Ranks | 1^v^ | 1.00 | 1.00 |
|  |  | Positive Ranks | 9^w^ | 6.00 | 54.00 |
|  |  | Ties | 0^x^ |  |  |
|  |  | Total | 10 |  |  |
|  | ClBW - ClPW | Negative Ranks | 0^y^ | .00 | .00 |
|  |  | Positive Ranks | 0^z^ | .00 | .00 |
|  |  | Ties | 10^aa^ |  |  |
|  |  | Total | 10 |  |  |

| **Ranks** | | | | | |
| --- | --- | --- | --- | --- | --- |
| Standard demineralizing solution (group 2) | | | N | Mean Rank | Sum of Ranks |
| 2 | ClBA - ClPA | Negative Ranks | 1^a^ | 1.00 | 1.00 |
|  |  | Positive Ranks | 0^b^ | .00 | .00 |
|  |  | Ties | 9^c^ |  |  |
|  |  | Total | 10 |  |  |
|  | KBW - KPW | Negative Ranks | 0^d^ | .00 | .00 |
|  |  | Positive Ranks | 0^e^ | .00 | .00 |
|  |  | Ties | 10^f^ |  |  |
|  |  | Total | 10 |  |  |
|  | KBA - KPA | Negative Ranks | 0^g^ | .00 | .00 |
|  |  | Positive Ranks | 0^h^ | .00 | .00 |
|  |  | Ties | 10^i^ |  |  |
|  |  | Total | 10 |  |  |
|  | CaBW - CaPW | Negative Ranks | 1^j^ | 4.00 | 4.00 |
|  |  | Positive Ranks | 9^k^ | 5.67 | 51.00 |
|  |  | Ties | 0^l^ |  |  |
|  |  | Total | 10 |  |  |
|  | CaBA - CaPA | Negative Ranks | 1^m^ | 2.00 | 2.00 |
|  |  | Positive Ranks | 9^n^ | 5.89 | 53.00 |
|  |  | Ties | 0^o^ |  |  |
|  |  | Total | 10 |  |  |
|  | NBW - NPW | Negative Ranks | 9^p^ | 5.89 | 53.00 |
|  |  | Positive Ranks | 1^q^ | 2.00 | 2.00 |
|  |  | Ties | 0^r^ |  |  |
|  |  | Total | 10 |  |  |
|  | NBA - NPA | Negative Ranks | 9^s^ | 5.89 | 53.00 |
|  |  | Positive Ranks | 1^t^ | 2.00 | 2.00 |
|  |  | Ties | 0^u^ |  |  |
|  |  | Total | 10 |  |  |
|  | SBW - SPW | Negative Ranks | 0^v^ | .00 | .00 |
|  |  | Positive Ranks | 0^w^ | .00 | .00 |
|  |  | Ties | 10^x^ |  |  |
|  |  | Total | 10 |  |  |
|  | SBA - SPA | Negative Ranks | 0^y^ | .00 | .00 |
|  |  | Positive Ranks | 0^z^ | .00 | .00 |
|  |  | Ties | 10^aa^ |  |  |
|  |  | Total | 10 |  |  |

| **Ranks** | | | | | |
| --- | --- | --- | --- | --- | --- |
|  | | | N | Mean Rank | Sum of Ranks |
| 2 | PRB - PRP | Negative Ranks | 7^a^ | 5.29 | 37.00 |
|  |  | Positive Ranks | 2^b^ | 4.00 | 8.00 |
|  |  | Ties | 1^c^ |  |  |
|  |  | Total | 10 |  |  |
|  | VHNB - VHNP | Negative Ranks | 1^d^ | 2.00 | 2.00 |
|  |  | Positive Ranks | 9^e^ | 5.89 | 53.00 |
|  |  | Ties | 0^f^ |  |  |
|  |  | Total | 10 |  |  |

**[ Abbreviations:** *CBW: Carbon Baseline Weight %; CBA: Carbon Baseline Atomic %; CPW: Carbon Post Exposure Weight %; CPA: Carbon Post Exposure Atomic %; OBW: Oxygen Baseline Weight %; OBA: Oxygen Baseline Atomic %; OPW:Oxygen Post Exposure Weight %; OPA: Oxygen Post Exposure Atomic %; FBW: Fluoride Baseline Weight %; FBA: Fluoride Baseline Atomic %; FPW: Fluoride Post Exposure Weight %; FPA: Fluoride Post Exposure Atomic %; NaBW: Sodium Baseline Weight %; NaBA: Sodium Baseline Atomic % ; NaPW: Sodium Post Exposure Weight %; NaPA: Sodium Post Exposure Atomic %; MgBW: Magnesium Baseline Weight %; MgBA: Magnesium Baseline Atomic %; MgPW: Magnesium Post Exposure Weight %; MgPA : Magnesium Post Exposure Atomic %; AlBW: Aluminium Baseline Weight %; AlBA: Aluminium Baseline Atomic %; AlPW: Aluminium Post Exposure Weight %; AlPA: Aluminium Post Exposure Atomic %; SiBW: Silicon Baseline Weight %; SiBA: Silicon Baseline Atomic %; SiPW: Silicon Post Exposure Weight %; SiPA: Silicon Post Exposure Atomic %; PBW: Phosphorus Baseline Weight %; PBA: Phosphorus Baseline Atomic %; PPW: Phosphorus Post Exposure Weight %; PPA: Phosphorus Post Exposure Atomic %; ClBW: Chlorine Baseline Weight %; ClBA: Chlorine Baseline Atomic %; ClPW; Chlorine Post Exposure Weight %; ClPA: Chlorine Post Exposure Atomic %; KBW : Potassium Baseline Weight %; KBA: Potassium Baseline Atomic %; KPW: Potassium Post Exposure Weight %; KPA: Potassium Post Exposure Atomic %; CaBW: Calcium Baseline Weight %; CaBA: Calcium Baseline Atomic %; CaPW: Calcium Post Exposure Weight %; CaPA: Calcium Post Exposure Atomic %; NBW: Nitrogen Baseline Weight %; NBA: Nitrogen Baseline Atomic %; NPW: Nitrogen Post Exposure Weight %; NPA: Nitrogen Post Exposure Atomic %; SBW: Sulphur Baseline Weight %; SBA: Sulphur Baseline Atomic %; SPW: Sulphur Post Exposure Weight %; SPA: Sulphur Post Exposure Atomic %; PRB: Profilometer Baseline: PRP: Profilometer Post Exposure; VHNB Vicker's Hardness Baseline; VHNP: Vicker's Hardness Post]*

**Supplementary Table 2: Comparison of the probiotic solution to standard demineralizing agent using Mann-Whitney Test**

| **Ranks** | | | | |
| --- | --- | --- | --- | --- |
|  | V1 | N | Mean Rank | Sum of Ranks |
| CW | 1 | 10 | 12.15 | 121.50 |
|  | 2 | 10 | 8.85 | 88.50 |
|  | Total | 20 |  |  |
| CA | 1 | 10 | 12.40 | 124.00 |
|  | 2 | 10 | 8.60 | 86.00 |
|  | Total | 20 |  |  |
| OW | 1 | 10 | 10.60 | 106.00 |
|  | 2 | 10 | 10.40 | 104.00 |
|  | Total | 20 |  |  |
| OA | 1 | 10 | 9.45 | 94.50 |
|  | 2 | 10 | 11.55 | 115.50 |
|  | Total | 20 |  |  |
| FW | 1 | 10 | 8.80 | 88.00 |
|  | 2 | 10 | 12.20 | 122.00 |
|  | Total | 20 |  |  |
| FA | 1 | 10 | 8.80 | 88.00 |
|  | 2 | 10 | 12.20 | 122.00 |
|  | Total | 20 |  |  |
| NW | 1 | 10 | 10.00 | 100.00 |
|  | 2 | 10 | 11.00 | 110.00 |
|  | Total | 20 |  |  |
| NA | 1 | 10 | 11.00 | 110.00 |
|  | 2 | 10 | 10.00 | 100.00 |
|  | Total | 20 |  |  |
| MgW | 1 | 10 | 10.50 | 105.00 |
|  | 2 | 10 | 10.50 | 105.00 |
|  | Total | 20 |  |  |
| MgA | 1 | 10 | 10.50 | 105.00 |
|  | 2 | 10 | 10.50 | 105.00 |
|  | Total | 20 |  |  |
| AlW | 1 | 10 | 12.25 | 122.50 |
|  | 2 | 10 | 8.75 | 87.50 |
|  | Total | 20 |  |  |
| AlA | 1 | 10 | 10.90 | 109.00 |
|  | 2 | 10 | 10.10 | 101.00 |
|  | Total | 20 |  |  |

| **Ranks** | | | | |
| --- | --- | --- | --- | --- |
|  | V1 | N | Mean Rank | Sum of Ranks |
| SiW | 1 | 10 | 11.50 | 115.00 |
|  | 2 | 10 | 9.50 | 95.00 |
|  | Total | 20 |  |  |
| SiA | 1 | 10 | 11.00 | 110.00 |
|  | 2 | 10 | 10.00 | 100.00 |
|  | Total | 20 |  |  |
| PW | 1 | 10 | 8.85 | 88.50 |
|  | 2 | 10 | 12.15 | 121.50 |
|  | Total | 20 |  |  |
| PA | 1 | 10 | 8.80 | 88.00 |
|  | 2 | 10 | 12.20 | 122.00 |
|  | Total | 20 |  |  |
| ClW | 1 | 10 | 10.60 | 106.00 |
|  | 2 | 10 | 10.40 | 104.00 |
|  | Total | 20 |  |  |
| ClA | 1 | 10 | 11.00 | 110.00 |
|  | 2 | 10 | 10.00 | 100.00 |
|  | Total | 20 |  |  |
| KW | 1 | 10 | 11.50 | 115.00 |
|  | 2 | 10 | 9.50 | 95.00 |
|  | Total | 20 |  |  |
| KA | 1 | 10 | 10.50 | 105.00 |
|  | 2 | 10 | 10.50 | 105.00 |
|  | Total | 20 |  |  |
| CaW | 1 | 10 | 8.80 | 88.00 |
|  | 2 | 10 | 12.20 | 122.00 |
|  | Total | 20 |  |  |
| CaA | 1 | 10 | 8.80 | 88.00 |
|  | 2 | 10 | 12.20 | 122.00 |
|  | Total | 20 |  |  |
| NW | 1 | 10 | 9.35 | 93.50 |
|  | 2 | 10 | 11.65 | 116.50 |
|  | Total | 20 |  |  |
| NA | 1 | 10 | 8.35 | 83.50 |
|  | 2 | 10 | 12.65 | 126.50 |
|  | Total | 20 |  |  |

| **Ranks** | | | | |
| --- | --- | --- | --- | --- |
|  | V1 | N | Mean Rank | Sum of Ranks |
| SW | 1 | 10 | 10.50 | 105.00 |
|  | 2 | 10 | 10.50 | 105.00 |
|  | Total | 20 |  |  |
| SA | 1 | 10 | 10.50 | 105.00 |
|  | 2 | 10 | 10.50 | 105.00 |
|  | Total | 20 |  |  |

**Changes in the pH of the probiotic and demineralizing solution with time [group- probiotics; group 2: demineralizing agent (0.1 M lactic acid)**

| **Time interval / Mean pH** | **0 hr** | | **2 hrs** | | **4 hrs** | | **6 hrs** | | **8 hrs** | | **12 hrs** | | **24 hrs** | |
| --- | --- | --- | --- | --- | --- | --- | --- | --- | --- | --- | --- | --- | --- | --- |
| **groups** | **Group 1** | **Group 2** | **Group 1** | **Group 2** | **Group 1** | **Group 2** | **Group 1** | **Group 2** | **Group 1** | **Group 2** | **Group 1** | **Group 2** | **Group 1** | **Group 2** |
| **1** | **5.80** | **4.80** | **4.75** | **4.88** | **4.49** | **4.70** | **3.90** | **4.26** | **3.61** | **4.26** | **3.26** | **4.16** | **3.10** | **4.00** |
| **2** | **5.77** | **4.97** | **4.74** | **4.98** | **4.47** | **3.68** | **3.89** | **4.27** | **3.55** | **4.27** | **3.21** | **4.17** | **3.09** | **4.02** |
| **3** | **5.76** | **4.83** | **4.74** | **4.90** | **4.47** | **4.89** | **4.01** | **4.28** | **3.56** | **4.30** | **3.11** | **4.10** | **2.98** | **3.89** |
| **Mean** | **5.78** | **4.86** | **4.74** | **4.92** | **4.48** | **4.42** | **3.93** | **4.27** | **3.57** | **4.27** | **3.19** | **4.14** | **3.06** | **3.95** |
